# Antibiofilm and Antivirulence Potentials of 3,2′-Dihydroxyflavone against *Staphylococcus aureus*

**DOI:** 10.3390/ijms25158059

**Published:** 2024-07-24

**Authors:** Inji Park, Yong-Guy Kim, Jin-Hyung Lee, Jintae Lee

**Affiliations:** School of Chemical Engineering, Yeungnam University, Gyeongsan 38541, Republic of Korea; pij3360@ynu.ac.kr (I.P.); yongguy7@ynu.ac.kr (Y.-G.K.)

**Keywords:** biofilm, *Candida albicans*, 3,2′-dihydroxyflavone, *Staphylococcus aureus*

## Abstract

*Staphylococcus aureus*, particularly drug-resistant strains, poses significant challenges in healthcare due to its ability to form biofilms, which confer increased resistance to antibiotics and immune responses. Building on previous knowledge that several flavonoids exhibit antibiofilm activity, this study sought to identify a novel flavonoid capable of effectively inhibiting biofilm formation and virulence factor production in *S. aureus* strains including MRSA. Among the 19 flavonoid-like compounds tested, 3,2′-dihydroxyflavone (3,2′-DHF) was identified for the first time as inhibiting biofilm formation and virulence factors in *S. aureus* with an MIC 75 µg/mL. The antibiofilm activity was further confirmed by microscopic methods. Notably, 3,2′-DHF at 5 µg/mL was effective in inhibiting both mono- and polymicrobial biofilms involving *S. aureus* and *Candida albicans*, a common co-pathogen. 3,2′-DHF reduces hemolytic activity, slime production, and the expression of key virulence factors such as hemolysin gene *hla* and nuclease gene *nuc1* in *S. aureus*. These findings highlight the potential of 3,2′-DHF as a novel antibiofilm and antivirulence agent against both bacterial and fungal biofilms, offering a promising alternative to traditional antibiotics in the treatment of biofilm-associated infections.

## 1. Introduction

*Staphylococcus aureus* is a principal pathogen in nosocomial and community-acquired infections, renowned for its ability to form biofilms. These biofilms substantially enhance bacterial resistance to antimicrobial therapies and the host immune system, complicating treatment strategies and contributing to chronic infections [1]. Traditional antibiotics are often ineffective against drug-resistant *S. aureus* such as methicillin and vancomycin, resistant *S. aureus* strains (MRSA and VRSA) as well as biofilm-associated infections due to the inherent resistance conferred by the biofilm matrix [2]. Furthermore, its ability to affect a wide range of tissues is due to its arsenal of virulence factors, which include adhesins, alpha-toxin (Hla), enterotoxins, enzymes, and staphyloxanthin [3]. These virulence factors are regulated by global regulatory systems such as the Agr system, SarA (staphylococcal accessory regulator), and the SaeRS two-component system [3]. The expression of these virulence factors makes *S. aureus* a versatile and formidable pathogen, complicating treatments, especially in the presence of antibiotic resistance. Therefore, innovative approaches that can diminish biofilm formation and virulence factor production are crucial for advancing clinical therapeutics.

Flavonoids are a diverse group of plant-derived polyphenolic compounds known for their potent antibacterial and anti-inflammatory properties. Flavonoids have also previously demonstrated considerable promise as antibiofilm agents against *S. aureus* strains. Research across multiple studies has highlighted their dual role in inhibiting biofilm formation and reducing virulence without affecting bacterial viability, offering a strategic advantage over traditional antibiotics by potentially reducing the likelihood of resistance development. Recently, several reviews introduced the anti-virulence potentials of plant flavonoids against *S. aureus* [4,5,6]. For example, several flavonoids including apigenin, kaempferol, luteolin, and quercetin have been shown to significantly diminish *S. aureus* biofilm formation and hemolytic activity [7,8,9,10]. Recently, our research has focused on the antibiofilm properties of various flavonoids against *Vibrio* species [11].

Building on previous works, the present study aimed to evaluate the antibiofilm effects of 19 flavonoid-like compounds encompassing flavones (apigenin, 7-hydroxyflavone, chrysin, 6-hydroxyflavone, 6-aminoflavone, 7,2-dihydroxyflavone, and quercetin), flavonols (epicatechin, catechin, and fisetin), flavonones (flavanone and naringin), and isoflavonoids (daidzein and genistein) on *S. aureus* including MRSA strains and to explore the underlying mechanisms responsible for these effects. After the initial screening, the novel active 3,2′-dihydroxyflavone (3,2′-DHF) was selected, and its activity was then compared with gentamicin in *S. aureus*. Its antibiofilm activity was also investigated against mixed biofilms of *S. aureus* and *C. albicans.* Live imaging microscopy, scanning electron microscopy, slime production, hemolytic activity, and qRT-PCR were used to investigate how 3,2′-DHF affects biofilm formation and toxin production in *S. aureus*.

## 2. Results

### 2.1. Antimicrobial and Antibiofilm Activity of Various Flavonoids against S. aureus

**Figure 1:**
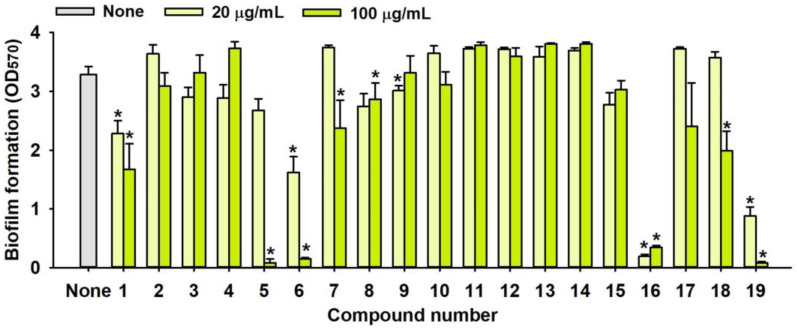
The antibiofilm screening of 19 flavonoid-like compounds. Biofilm formation by *S. aureus* ATCC 6538 with flavonoids at 20 or 100 µg/mL in 96-well polystyrene plates after 24 h culture. * Denotes a significant difference at *p* < 0.05 and the error bars represent the standard deviation.

The biofilm inhibitory capabilities of 19 flavonoid-like compounds against the *S. aureus* MSSA 6538 strain were initially tested at concentrations of 20 and 100 µg/mL, as detailed in Figure 1. At 100 µg/mL, 3,2′-dihydroxyflavone (3,2′-DHF) (5), curcumin (6), quercetin (16), and fisetin (19) reduced *S. aureus* biofilm formation by over 90%. Additionally, the minimum inhibitory concentrations (MICs) of these effective compounds were determined to gauge their antibacterial activity. Specifically, 3,2′-dihydroxyflavone (3,2′-DHF), curcumin, quercetin, and fisetin completely inhibited the planktonic cell growth of *S. aureus* at concentrations of 75, 50, 400, and 200 µg/mL, respectively (Table 1). The findings suggest that the antibiofilm effects of 3,2′-dihydroxyflavone and curcumin are predominantly due to their antibacterial properties, whereas quercetin’s biofilm inhibition at sub-MIC levels is not solely dependent on growth inhibition. Although the antibiofilm activities of curcumin, quercetin, and fisetin have been previously documented [7,8,9,10], this study marks the first report of the antimicrobial and antibiofilm activity of 3,2′-DHF against *S. aureus*. Consequently, 3,2′-DHF was chosen for further investigation for its antibiofilm and antivirulence activities and compared with the activities of antibiotic gentamicin.

**Figure 2:**
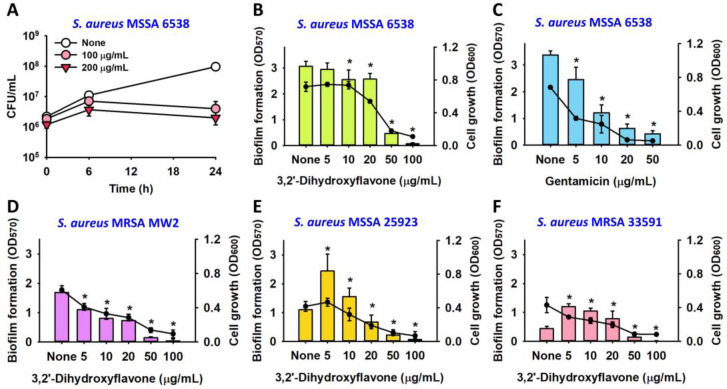
Effects of 3,2′-DHF on the *S. aureus* biofilm and planktonic cell growth. CFU measurement with 3,2′-DHF (**A**). Biofilm inhibition of *S. aureus* ATCC 6538 with 3,2′-DHF (**B**) and gentamicin (**C**) in 96-well polystyrene plates after 24 h culture. Biofilm inhibition of MRSA MW2 (**D**), MSSA 25923 (**E**), and MRSA 33591 (**F**). * *p* < 0.05 vs. non-treated controls (none). Bar graphs represent biofilm formation, while line graphs depict cell growth (**B**–**F**).

The antimicrobial and antibiofilm activities of 3,2′-DHF were explored in more detail using additional *S. aureus* strains. 3,2′-DHF demonstrated a dose-dependent inhibition of planktonic cell growth, with an MIC of 50–75 µg/mL observed across all of the tested strains including MSSA 6538, MSSA 25923, MRSA 33591, and MRSA MW2 (Figure S1). A time-kill kinetic study was conducted to evaluate the bacteriostatic or bactericidal effects of 3,2′-DHF against *S. aureus*. The results indicated that 3,2′-DHF acts in a bacteriostatic manner, maintaining 10^6^ cells with 200 µg/mL of 3,2′-DHF treatment after 24 h (Figure 2A).

While the antibacterial activities of 3,2′-DHF were consistent across four *S. aureus* strains, its antibiofilm effects varied. 3,2′-DHF dose-dependently inhibited biofilm formation in the MSSA 6538 and MRSA MW2 strains, akin to the effects of gentamicin, attributable to its inhibition of planktonic growth (Figure 2A–D). However, for the MSSA 25923 and MRSA 33591 strains, 3,2′-DHF at sub-MIC levels (5–10 µg/mL) significantly increased biofilm formation, whereas near-MIC levels (50–100 µg/mL) reduced it (Figure 2E,F). These observations align with prior studies indicating that many antimicrobial agents can induce microbial biofilm formation at sub-MICs as part of a microbial defense mechanism [12], although the specific underlying mechanisms remain to be elucidated.

### 2.2. Observation of the Antibiofilm Effects of 3,2′-DHF

**Figure 3:**
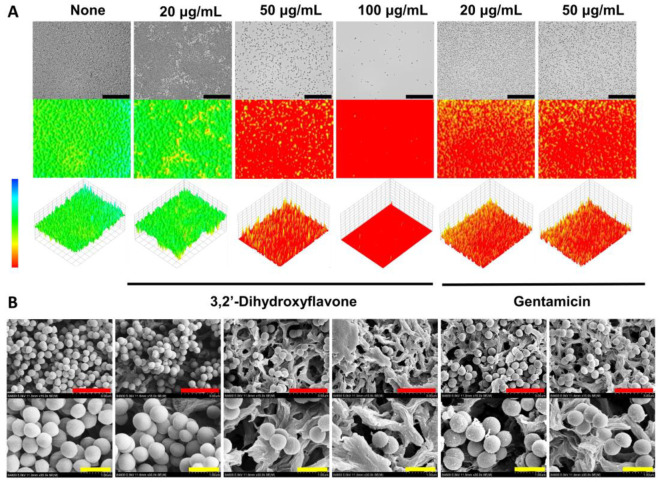
Microscopic observation of *S. aureus* biofilm inhibition. Live microscopy 2D and 3D images of *S. aureus* (**A**), and SEM images of *S. aureus* ATCC 6538 treated with 3,2′-DHF and gentamicin (**B**). The black, red, and yellow scale bar represent 50, 3, and 1 μm, respectively.

The antibiofilm potentials of 3,2′-DHF and gentamicin were assessed using live microscopy and SEM. Both 2D and 3D microscopic imaging revealed that 3,2′-DHF at concentrations of 50 or 100 µg/mL significantly prevented biofilm formation compared to the dense biofilms in the untreated control, similar to the effects seen with gentamicin at 20 or 50 µg/mL (Figure 3A). SEM analysis further confirmed the antibiofilm activities of both 3,2′-DHF and gentamicin, showing a reduced number of cells in the treated samples compared to the untreated control, while not affecting the morphology of *S. aureus* cells (Figure 3B).

### 2.3. Antibiofilm Effect of 3,2′-DHF on Dual Biofilms of S. aureus and C. albicans

**Figure 4:**
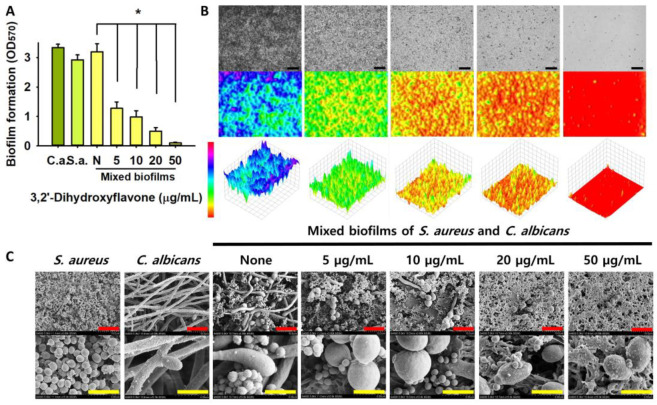
Effects of 3,2′-DHF on dual biofilms of *S. aureus* and *C. albicans*. Biofilm formation by *S. aureus* ATCC 6538 and *C. albicans* DAY185 with 3,2′-DHF in 96-well polystyrene plates after 24 h culture. C.a. and S.a. represent *C. albicans* and *S. aureus*, respectively. N represents none treated contrrol (**A**). Live microscopy 2D and 3D images of *S. aureus* and *C. albicans* (**B**). SEM images of dual biofilms treated with 3,2′-DHF (**C**). The black, red, and yellow scale bar represent 100, 10, and 3 μm, respectively. * *p* < 0.05 vs. non-treated controls (none).

*S. aureus* and *C. albicans* often form polymicrobial biofilms that display increased resistance to antimicrobial agents [13]. Our group recently found the antibiofilm activity of 3,2′-DHF against *Candida albicans* strains. Building on this, we assessed the inhibitory efficacy of 3,2′-DHF against mixed biofilms of S. *aureus* and *C. albicans*. Consistent with our expectations, 3,2′-DHF at concentrations above 5 μg/mL dose-dependently inhibited the biofilm formation of both species (Figure 4A).

As expected, 2D and 3D microscopic analysis showed that 3,2′-DHF (5–50 µg/mL) inhibited the dual biofilms of *S. aureus* and *C. albicans* (Figure 4B), and SEM analysis further confirmed the inhibitory impact of 3,2′-DHF on dual biofilm formation (Figure 4C). The untreated control displayed large *C. albicans* hyphal filaments intertwined with dense clusters of smaller *S. aureus* cells within the biofilm matrix. Treatment with 3,2′-DHF at 5–20 μg/mL effectively eliminated noticeable hyphal filaments, although some *S. aureus* cells remained visible. Increasing the concentration of 3,2′-DHF to 50 μg/mL effectively eliminated most cells from both species. This suggests that the *S. aureus* biofilm exhibited greater resistance to 3,2′-DHF compared to the *C. albicans* biofilm.

### 2.4. Effects of 3,2′-DHF on Slime Production and Hemolytic Activity in S. aureus

**Figure 5:**
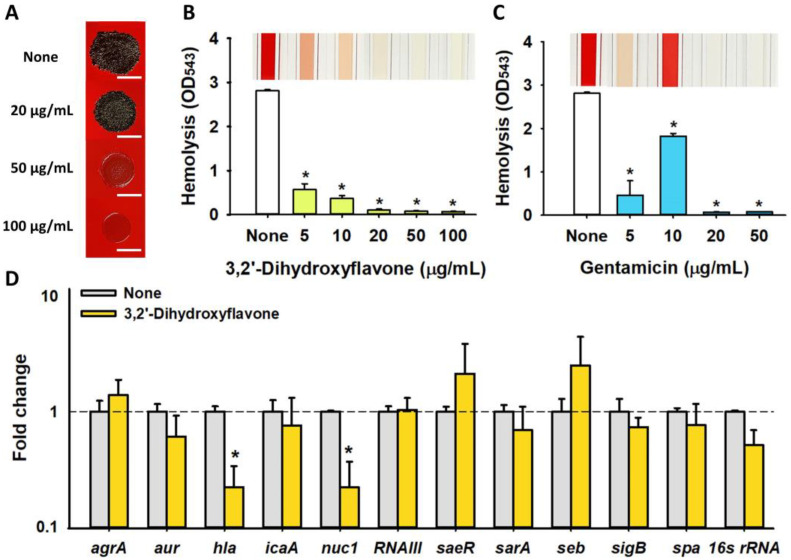
Effect of 3,2′-DHF on *S. aureus* virulence factors. Slime production (**A**). Black color indicates slime production on the Congo Red agar plates. Hemolytic activity of 3,2′-DHF (**B**) and gentamicin (**C**). The effect of 3,2′-DHF (50 µg/mL) on the gene expression in *S. aureus* ATCC 6538. *16s rRNA* was the housekeeping gene (**D**). * *p* < 0.05 vs. untreated controls (none). The white scale bar represents 500 μm.

*S. aureus* produces slime, which is pivotal for its biofilm formation and is closely associated with its pathogenicity [14]. Hence, the effect of 3,2′-DHF on slime production in MSSA 6538 was investigated. 3,2′-DHF inhibited slime production in a dose-dependent manner; notably, concentrations of 50 or 100 μg/mL completely abolished slime production, primarily through the inhibition of bacterial growth (Figure 5A).

Hemolytic activity, driven by alpha-hemolysin, is a key virulence factor in *S. aureus* [15]. Alpha-toxin, encoded by the *hla* gene, has the capability to lyse red blood cells. We evaluated the effects of 3,2′-DHF and gentamicin on the hemolytic ability of MSSA 6538. 3,2′-DHF was found to dose-dependently inhibit hemolytic activity, with concentrations as low as 5 μg/mL reducing the activity by more than 79% (Figure 5B). In contrast, gentamicin displayed a biphasic effect on hemolytic activity, indicating a variable response at different concentrations (Figure 5C).

### 2.5. Differential Gene Expression Induced by 3,2′-DHF in S. aureus

To study the mechanisms of the antibiofilm and antivirulence effects of 3,2′-DHF on *S. aureus*, qRT-PCR was performed to assess the expressions of 11 biofilm- and toxin-related genes as well as the global regulatory genes in *S. aureus* MSSA 6538 cells. Treatment with 3,2′-DHF at a concentration of 50 µg/mL led to a significant downregulation of *hla* (alpha-toxin) and *nuc1* (staphylococcal nuclease) while the expression levels of the other genes (*agrA*, *aur*, *icaA*, *RNAIII*, *saeR*, *sarA*, *sigB*, and *spa*) remained unchanged (Figure 5D). Notably, the suppression of *hla* expression by 3-fold is consistent with the observed reduction in hemolytic activity (Figure 5B), highlighting the specific antivirulence action of 3,2′-DHF.

## 3. Discussion

The current study reports on the antimicrobial and antibiofilm effects of various flavonoids against *S. aureus*, and partially revealed the mechanisms of the most active compound 3,2′-DHF. While the antimicrobial and antibiofilm activities of flavonoids have been widely reported, this is the first report of 3,2′-DHF’s effect on *S. aureus* and on dual-species biofilms with *C. albicans*.

3,2′-DHF was found in the climbing plant *Marsdenia tinctoria* [16]. Previously, its beneficial effects have been reported on skin regeneration [17] and embryonic stem cell proliferation [18,19]. Additionally, a combination of quercetin and 3,2′-DHF has been used to enhance the proliferation and differentiation of porcine muscle stem cells in cultured meat processes [20], and the antioxidant properties of hydroxyflavones are well-documented [21].

3,2′-DHF exhibited an MIC of 75 µg/mL and at sub-inhibitory concentrations (5–20 µg/mL), exerted antibiofilm and anti-hemolysis activities against *S. aureus* (Figure 2 and Figure 5). The antibiofilm activity was partly due to the antimicrobial effect as well as the repression of hemolysin gene *hla* and nuclease gene *nuc1* in *S. aureus* (Figure 5D). Alpha-hemolysin (Hla) plays a positive role in biofilm formation by *S. aureus* [22], and previously, other flavonoids repressed the gene expression of *hla* and biofilm formation in *S. aureus* [7,15]. Hence, the current results support the previous findings. While *S. aureus* nuclease *nuc1* positively modulated biofilm formation and dispersal [23,24], 3,2′-DHF repressed the expression of *nuc1* (Figure 5D). This result suggests that biofilm reduction by 3,2′-DHF is less associated with *nuc1*.

Previously, several flavonoids such as quercetin [10] myricetin, hesperetin, scutellarein and phloretin [9,25] as well as naringenin [26,27] have displayed antibiofilm activity against *S. aureus*. The antimicrobial mechanism of flavonoids is closely related to cell membrane integrity in both Gram-negative and Gram-positive bacteria, although it remains controversial [28]. In the case of 3,2′-DHF, it exhibited bacteriostatic activity rather than bactericidal (Figure 2A) and there was no change in the cell membrane integrity after treatment with 3,2′-DHF (Figure 3B). Hence, it may not target the cell membrane, and identifying the key target genes or proteins in the future is important.

Among the 19 flavonoids tested, 3,2′-DHF, quercetin, and fisetin at 100 µg/mL demonstrated a complete inhibition of *S. aureus*, despite exhibiting weak antimicrobial activity (Figure 1). 3,2′-DHF, quercetin, and fisetin share similar structures including the hydroxyl group at the C3 position on the C-ring (Figure 1), which may be crucial to their antibiofilm activity. Recently, 3,2′-DHF also showed antimicrobial and antibiofilm activities against *Vibrio* spp. and *Salmonella typhimurium* [11], and even the *C. albicans* strain (Figure 4). Further investigation into compounds similar to 3,2′-DHF could lead to improved broad-spectrum antimicrobial activities.

3,2′-DHF inhibits slime production (Figure 5A), hemolytic activity (Figure 5B), and the expression of virulence factor genes (α-hemolysin *hla* and nuclease *nuc1*) (Figure 5D). Slime production by coagulase-negative *S. aureus* is considered as a virulence factor since slime enhances colonization and biofilm formation [29]. α-Hemolysin is a major toxin that causes blood hemolysis [30] and is known to upregulate the biofilm formation of *S. aureus* [22]. Additionally, the staphylococcal nuclease Nuc1 is a virulence factor that positively influences biofilm formation by modulating eDNA in the biofilm matrix [23]. Current results partially elucidate how 3,2′-DHF inhibits *S. aureus* biofilm formation and support the previous findings. Furthermore, 3,2′-DHF could also serve as a tool to reduce the pathogenesis of *S. aureus*.

## 4. Materials and Methods

### 4.1. Bacterial Strains, Growth Conditions, and Chemicals

This study utilized four *S. aureus* strains: two methicillin-sensitive *S. aureus* strains (MSSA; ATCC 6538 and ATCC 25923) and two MRSA strains (MRSA 33591 and MW2). All *S. aureus* strains were cultured in Luria-Bertani (LB) broth while two MRSA strains were cultured in LB additionally supplemented with 0.2% glucose at 37 °C. A fluconazole-resistant *C. albicans* DAY185 was cultured in potato dextrose broth (PDB) medium. Strains were acquired from the American Type Culture Collection (Manassas, VA, USA).

Nineteen flavonoids are shown in Figure 1, and gentamicin were purchased from Sigma-Aldrich (St. Louis, MO, USA). Dimethyl sulfoxide (DMSO) was used to dissolve the compounds and 0.1% (*v*/*v*) DMSO was used as a control, which had no effects on planktonic cell growth or biofilm formation. For planktonic cell growth assay, cell turbidity and colony-forming units (CFUs) were measured after culturing *S. aureus* cells in 96-well plates with or without flavonoids for 24 h. For the minimum inhibitory concentration (MIC) assay, the overnight culture of *S. aureus* was diluted (OD_600_ = 0.1 corresponding to ~10^7^ CFU) in LB medium with or without each flavonoid and cultured for 24 h before determining the cell growth. The MIC is the concentration where no planktonic cell growth was observed. The assay results were derived from at least two independent cultures conducted in triplicate.

### 4.2. Microtiter Dish Biofilm Formation Assay

The overnight culture of *S. aureus* was diluted (~10^7^ cells) in LB from two MSSA strains and LB with 0.2% glucose medium for two MRSA strains with flavonoids (0, 5, 10, 20, 50, or 100 µg/mL) or gentamicin (0, 5, 10, 20, or 50 µg/mL). Samples of 300 µL were then placed into 96-well polystyrene plates (SPL Life Sciences, Pocheon, Republic of Korea) and incubated without agitation for 24 h at 37 °C. Post-incubation, planktonic cell growth was assessed by measuring optical density at 620 nm (OD_620_) using a Multiskan EX microplate reader (Thermo Fisher Scientific, Waltham, MA, USA). To quantify the biofilm formation, the supernatant containing planktonic cells was discarded, and the plates were washed three times with distilled water. Biofilm cells were dyed with crystal violet (0.1%) for 20 min, rinsed with distilled water, and the stain was solubilized in 95% ethanol. The optical densities of the solution were measured at 570 nm (OD_570_) using the Multiskan EX microplate reader. Results are presented as the means derived from at least six repetitions across two independent cultures [31].

### 4.3. Time–Kill Kinetics Assay

The bactericidal or bacteriostatic effects of 3,2′-DHF were assessed with minor modifications [32]. An overnight culture of *S. aureus* was inoculated (~10^7^ cells) into 2 mL tubes with or without 3,2′-DHF at 100 µg/mL or 200 µg/mL. The samples were then incubated at 37 °C with shaking at 250 rpm. At 0, 6, and 24 h, 100 µL samples were taken, serially diluted, and spread on LB agar plates, which were then incubated at 37 °C. Colony-forming units (CFUs) were counted post-incubation, and the results were reported as CFU/mL.

### 4.4. Biofilm Visualization by Live Microscopy and SEM

To observe the antibiofilm activity of 3,2′-DHF against *S. aureus*, biofilms of *S. aureus* MSSA 6538 were produced as above in 96-well plates for 24 h with 3,2′-DHF (0, 20, 50, or 100 µg/mL) or gentamicin (0, 20, or 50 µg/mL) at 37 °C. Subsequent to incubation, planktonic cells were removed by washing the wells three times with PBS buffer (pH 7.4). The biofilms were then imaged using the iRiS Digital Cell Imaging System (Logos BioSystems, Anyang, Korea). The captured images of the biofilms were processed into 2D and 3D color-coded visual representations using ImageJ 1.53k software [33].

The SEM study was conducted according to an established procedure [33]. Briefly, 300 μL of diluted *S. aureus* cells (~10^7^ cells CFU/mL) with 3,2′-DHF (0, 20, 50 or 100 µg/mL) or gentamicin (0, 20, or 50 µg/mL) were dispensed into 96-well plates, each containing a sterile nylon filter membrane (0.4 × 0.4 mm). The plates were incubated for 24 h at 37 °C without agitation. After incubation, the biofilms that had formed on the membranes were fixed with a mixture of 2% formaldehyde and 2.5% glutaraldehyde for 24 h. The biofilms were then dehydrated in a gradient series of ethanol concentrations. Following critical-point drying using an HCP-2 apparatus (Hitachi, Tokyo, Japan) and platinum sputter-coating, the samples were examined under an S-4800 scanning electron microscope (Hitachi, Tokyo, Japan) at 15 kV.

### 4.5. Biofilm Assay of Dual Species of S. aureus and C. albicans

To assess multispecies biofilm formation, we employed a method previously outlined in [34]. Briefly, *S. aureus* cells (5 × 10^6^ CFU/mL) and *C. albicans* cells (5 × 10^3^ CFU/mL) were co-inoculated into a mixed culture medium (LB/PDB = 1:1) in 96-well plates. The mixed cultures were then treated with 3,2′-DHF (0, 5, 10, 20, or 50 µg/mL) and incubated under static conditions at 37 °C for 24 h. Post-incubation, biofilm formation was assessed as previously described. Results are presented as the means derived from at least six repetitions across two independent cultures.

### 4.6. Slime Production Assay

Colony morphologies and slime production assays were conducted using Congo Red agar (CRA), as previously described [33]. The CRA consisted of brain-heart infusion broth (37 g/L), sucrose (36 g/L), agar (15 g/L), and Congo Red (0.8 g/L). Overnight cultures of *S. aureus* MSSA 6538 cells (10 μL) were dropped on CRA plates with 3,2′-DHF (0, 20, 50, or 100 µg/mL) and incubated for 24 h at 37 °C before imaging. The experiments were performed in duplicate. Black-colored colonies indicate substantial slime production, while pale-colored colonies signify an absence of slime.

### 4.7. Hemolytic Activity Assay

The anti-hemolytic activity of 3,2′-DHF or gentamicin was evaluated [33]. Briefly, 2  mL of diluted *S. aureus* cells (~10^7^ cells CFU/mL) in 14 mL tubes were treated with 3,2′-DHF (0, 20, 50 or 100 µg/mL) or gentamicin (0, 20, or 50 µg/mL) for 24 h with 250 rpm shaking. In parallel, sheep blood was centrifuged for 5 min at 4000 rpm, and the blood cells were washed three times with PBS buffer and diluted in PBS to a final concentration of 3.3% (*v*/*v*). Subsequently, 300 µL of the *S. aureus* culture was added to 1 mL aliquots of the diluted sheep blood and incubated with shaking at 250 rpm for 1 h at 37 °C. After incubation, the cells were pelleted by centrifugation for 10 min at 12,000 rpm, the supernatants were collected, and the optical densities of these supernatants were measured at 543 nm.

### 4.8. RNA Isolation and qRT-PCR

To assess changes in gene expression, a modified version of the previous transcriptomic assay was utilized [33]. *S. aureus* cells (~10^7^ cells CFU/mL) were inoculated into 25 mL LB medium in a 250 mL flask and incubated for 3 h at 37 °C with 250 rpm shaking. After this initial incubation, the culture was treated with or without 3,2′-DHF (50 µg/mL) at an optical density of 1.0 (OD_600_) and incubated for an additional 3 h. To preserve RNA integrity, cells were treated with an RNase inhibitor (RNAlater, Ambion, TX, USA) before being collected by centrifugation at 12,000 rpm for 10 min. For cell lysis, glass beads (150–212 μm, Sigma-Aldrich, ~10 times the volume of the cell pellet) were added to the lysis buffer. The mixture was then vigorously vortexed for 50 s and chilled on ice for 50 s between each vortex, then repeated twelve times to ensure thorough cell disruption. Following lysis, the supernatant was collected by centrifugation for 10 min at 13,000 rpm, and the total RNA was isolated using the Qiagen RNeasy MiniKit (Valencia, CA, USA). qRT-PCR was implemented using the SYBR™ Green qPCR Master Mix (Applied Biosystems, Foster City, CA, USA), the ABI StepOne Real-Time PCR System (Applied Biosystems), and primer sequences are listed in Table S1. Cycle threshold (Ct) values were obtained, and the 2^−ΔΔCT^ method was utilized to calculate the change in relative gene expression. 16S rRNA was used as an endogenous control, and the analysis was conducted with data from two independent cultures and four reactions per gene.

### 4.9. Statistical Analysis

All experiments were conducted using two independent cultures with two or three replicates each, and the results are presented as means ± standard deviations (SDs). Statistical significance was calculated using the Student’s *t*-test, with differences considered significant at *p* < 0.05.

## 5. Conclusions

The current findings suggest that 3,2′-DHF could be effective in treating *S. aureus*-associated skin infections due to its antimicrobial, antibiofilm, and antivirulence activities. Notably, 3,2′-DHF showed broad antibiofilm potential against *S. aureus* and *C. albicans*. Further molecular studies to identify its targets (genes or proteins), along with in vivo and toxicological studies, are necessary to confirm its efficacy and safety in clinical settings.

## Figures and Tables

**Table 1:** Full chemical names and structures corresponding to the numbers.

No.	Material	Structure	MIC (μg/mL)	No.	Material	Structure	MIC (μg/mL)
1	Apigenin	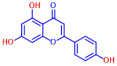	>400	11	Flavanone	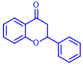	>400
2	7-Hydroxyflavone	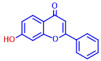	>400	12	6-Hydroxyflavone	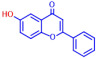	>400
3	Epicatechin	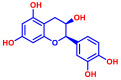	>400	13	6-Aminoflavone	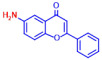	>400
4	Catechin	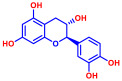	>400	14	Flavone	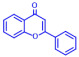	>400
5	3,2’-Dihydroxyflavone	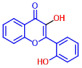	75	15	Naringin	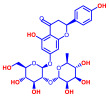	>400
6	Curcumin	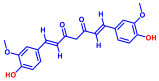	50	16	Quercetin	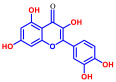	>400
7	2,2’-Dihydroxy-4-methoxybenzophenone	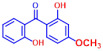	200	17	Genistein	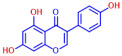	>400
8	2,2’-Dihydroxy-4,4’-dimethoxybenzophenone	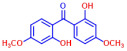	>400	18	Phloretin	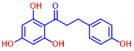	>400
9	Daidzein	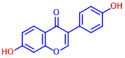	>400	19	Fisetin	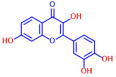	200
10	Chrysin	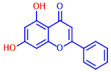	>400				

## Data Availability

Data is contained within the article and Supplementary Materials.

## Supplementary Materials

The following supporting information can be downloaded at: https://www.mdpi.com/article/10.3390/ijms25158059/s1.
