# Supplementary material for: Antibiofilm and Antivirulence Potentials of 3,2′-Dihydroxyflavone against Staphylococcus aureus
Source: Int J Mol Sci. 2024 Jul 24;25(15):8059. doi: 10.3390/ijms25158059 (PMC11311418; doi:10.3390/ijms25158059)
Supplement: Supplementary file 1 [file ijms-25-08059-s001.zip › ijms-3093017-supplementary.pdf]

Supplementary Materials

Antibiofilm and antivirulence potentials of 3,2'-dihydroxyflavone against *Staphylococcus aureus*

Inji Park [1,†], Yong-Guy Kim [1,†], Jin-Hyung Lee [1,*], and Jintae Lee [1,*]

*Correspondence: jinhlee@ynu.ac.kr; jtlee@ynu.ac.kr; Tel.: +82-53-810-2533

Table S1. Sequences of the primers used for quantitative RT-PCR.

Gene	Name	Primer
16S rRNA	A component of ribosomes	Forward 5'-TGT TTG ACG ATG TTT GAG CA-3'<br>Reverse 5'-CCT TCC TCC AGT TCA GAT GC -3'
agrA	Quorum-sensing regulator A	Forward 5'-TGA TAA TCC TTA TGA GGT GCT T-3'<br>Reverse 5'-CAC TGT GAC TCG TAA CGA AAA-3'
aur	Zinc metalloproteinase aureolysin	Forward 5'-ACC GTG TGT TAA TTC GTG TGC TA-3'<br>Reverse 5'-ATG GTC GCA CAT TCA CAA GTT T-3'
hla	α-Hemolysin	Forward 5'-CGG CAC ATT TGC ACC AAT AAG GC-3'<br>Reverse 5'-GGT TTA GCC TGG CCT TCA GC-3'
icaA	Intercellular adhesion A	Forward 5'-TGA ACC GCT TGC CAT GTG-3'<br>Reverse 5'-CAC GCG TTG CTT CCA AAG A-3'
nuc1	Nuclease	Forward 5'-CAC CTG AAA CAA AGC ATC CTA A-3'<br>Reverse 5'-TAT ACG CTA AGC CAC GTC CAT-3'<br>Reverse 5'-TTG TGA ATT TTT CTT CTT CGG ACA-3'
RNA III	Transcriptional regulator	Forward 5'-ATC GAC ACA GTG AAC AAA TTC AC-3'<br>Reverse 5'-CTC TAC TAG CAA ATG TTA CTC AC-3'
saeR	Response regulator	Forward 5'-GCC TTA ACT TTA GGT GCA GAT GAC TAT GTC-3'<br>Reverse 5'-CGA CAG TTG TTC AAC TGG TTG ATG ATG G-3'
sarA	Transcriptional regulator	Forward 5'-GAG TTG TTA TCA ATG GTC-3'<br>Reverse 5'-GTT TGC TTC AGT GAT TCG-3'
seb	Enterotoxin B	Forward 5'-TGT TCG GGT ATT TGA AGA TGG -3'<br>Reverse 5'-CGT TTC ATA AGG CGA GTT GTT-3'
sigB	RNA Polymerase sigma factor	Forward 5'-AAG TGA TTC GTA AGG ACG TCT-3'<br>Reverse 5'-TCG ATA ACT ATA ACC AAA GCC T-3'
spa	Protein A	Forward 5'-ACC AGA AAC TGG TGA AGA AAA TCC-3'<br>Reverse 5'-TAA CGC TGC ACC TAA GGC TAA TG-3'

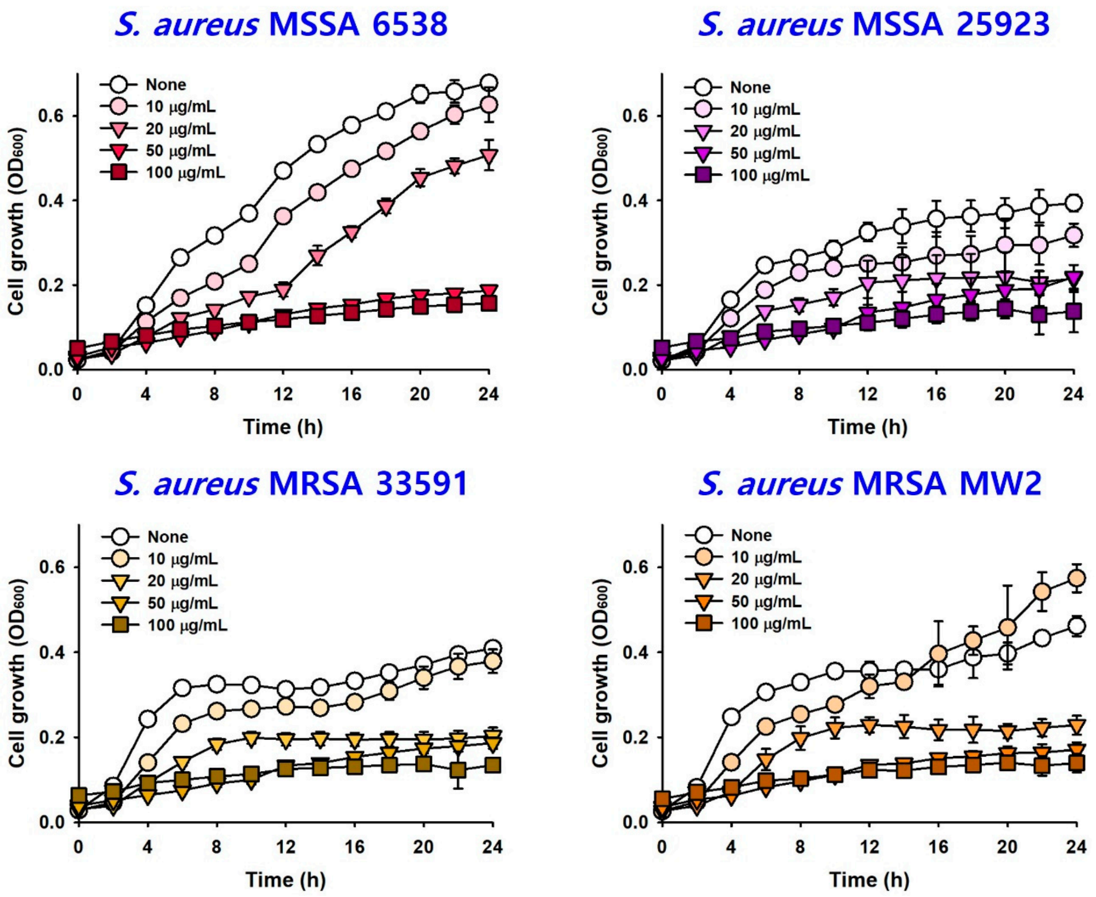

Figure S1. The effects of 3,2'-DHF on planktonic cell growth of other *S. aureus* strains.
